# Comparison of Various Rose Bengal Plate Tests, Serum Tube Agglutination Tests, and Enzyme-Linked Immunosorbent Assays Used in Brucellosis Serosurveillance in Sheep, Bovines, and Spotted Deer

**DOI:** 10.3390/vetsci13080804

**Published:** 2026-08-14

**Authors:** Weldeab Solomon Ghebrezgabher, Guodong Song, Jiazhen Ge, Yijian Liu, Pengcheng Gao, Renge Li, Fuying Zheng, Yuefeng Chu

**Affiliations:** 1 State Key Laboratory for Animal Disease Control and Prevention, College of Veterinary Medicine, Lanzhou University, Lanzhou Veterinary Research Institute, Chinese Academy of Agricultural Sciences, Lanzhou 730046, China; weldeabsolomon32@gmail.com (W.S.G.); songguodong166@163.com (G.S.); 18256589168@163.com (J.G.); lizibba@163.com (Y.L.); gaopengcheng@caas.cn (P.G.);; 2 National Higher Education and Research Institute, Hamelmalo Agricultural College, Keren P.O. Box 397, Eritrea; 3 College of Veterinary Medicine, Gansu Agricultural University, Lanzhou 730070, China; 4 Lanzhou Animal Disease Prevention and Control Center, Lanzhou 730050, China; 5 Gansu Province Research Center for Basic Disciplines of Pathogen Biology, Lanzhou 730046, China; 6 Key Laboratory of Veterinary Etiological Biology, Key Laboratory of Ruminant Disease Prevention and Control (West), Ministry of Agricultural and Rural Affairs, Lanzhou 730046, China

**Keywords:** brucellosis, comparison, ruminants, serological tests, serosurveillance

## Abstract

Brucellosis is a zoonotic disease causing significant economic losses, and necessitating standardized diagnostic kits for effective surveillance. The objectives of this study are to investigate brucellosis serosurveillance in ruminants, and to evaluate three serological tests—Rose Bengal Plate Tests (RBPTs), Serum Tube Agglutination Tests (STATs), and Enzyme-Linked Immunosorbent Assays (ELISAs)—each from three manufacturers. A total of 5797 serum samples (5260 sheep, 83 bovines, and 454 spotted deer) were initially screened with the RBPT, and 54 RBPT-positive selected samples were subsequently analyzed by the STAT and ELISA. RBPT screening showed significant variability: kit A (0.86%) showed significantly lower positivity than kits B and C (1.45% and 1.43%, *p* < 0.05). Among 54 RBPT-positive samples, STAT positivity from manufacturers A, B, and C was 61.11%, 70.37%, and 66.67%, respectively, with no significant differences (*p* > 0.017 in pairwise comparisons). All ELISAs consistently outperformed STATs. Kit B (competitive ELISA, 90.74%) had significantly higher positivity than kits A (indirect ELISA, 77.78%) and C (competitive ELISA, 74.07%) (*p* < 0.017 in pairwise comparisons). Key implications: Results from different manufacturers are not directly comparable, and concurrent use of different techniques on the same sample can yield discrepant outcomes. This underscores the urgent need for diagnostic kit standardization in brucellosis prevalence studies.

## Simple Summary

Brucellosis is a zoonotic bacterial disease that not only severely affects the health of both animals and humans but also causes significant economic losses, making accurate diagnosis essential for control and eradication. Proper diagnosis of brucellosis is important for the control and eradication of the disease in animals and, consequently, in humans. However, no definitive diagnostic technique exists, and diagnosis typically relies on a combination of two or more diagnostic methods. This study evaluated the positivity rates of three serological tests—RBPT, STAT, and ELISA—using reagents from three manufacturers (A, B, C) for each serological test. Among 5797 serum samples initially screened by the RBPT, positivity rates were 0.86% (A), 1.45% (B), and 1.43% (C), with A significantly lower than B and C (*p* < 0.05). Of 54 RBPT-positive samples subsequently retested by the STAT and ELISA, the STAT showed no significant manufacturer differences, but ELISA results varied significantly: kit B (c-ELISA) differed from kits B (i-ELISA) and C (c-ELISA) (*p* < 0.017 in pairwise comparisons). These discrepancies highlight the urgent need for standardization of *Brucella* serological test quality across manufacturers to improve detection accuracy, which ultimately supports better brucellosis disease control and eradication efforts.

## 1. Introduction

Brucellosis is a globally significant zoonotic disease caused by facultative intracellular, Gram-negative coccobacilli of the genus *Brucella*, posing substantial threats to both animal and human health with considerable economic consequences [1,2,3,4,5]. Brucellosis is recognized by the World Health Organization (WHO), Food and Agriculture Organization (FAO), and World Organization for Animal Health (WOAH) as one of the most prevalent zoonoses worldwide and ranks first among zoonotic bacterial diseases [6,7]. Although successfully eradicated in several industrialized nations, it remains endemic in many regions across Asia, Africa, the Middle East, and Latin America, continuing to challenge veterinary and public health systems globally [5,8,9].

In animals, brucellosis is transmitted horizontally by ingestion of organisms from contaminated feeds and water, mating, and direct contact with infected placentas and uterine discharges, through abraded skin, accidental injection, and inhalation of aerosolized bacteria [10,11], as well as vertically infecting newborn calves and lambs in the uterus [7,12]. It primarily manifests as a reproductive disease, causing abortion, stillbirth, infertility, orchitis, and reduced milk and meat production, resulting in significant economic losses to the livestock industry [4,13,14,15]. In humans, infection occurs through consumption of raw or unpasteurized dairy products, direct contact with infected animals, or inhalation of aerosolized bacteria, and may result in severe, debilitating illness requiring prolonged combination antibiotic therapy [7,14,16].

Accurate and reliable diagnosis of *Brucella* spp. infection is fundamental to disease control and eradication programs [17,18]. While bacterial culture remains the gold standard, its application is limited by biosafety requirements, lengthy processing times, and the occupational risk it poses to laboratory personnel [8,18,19,20]. Consequently, serological methods—including the Rose Bengal Plate Test (RBPT), Serum Tube Agglutination Test (STAT), complement fixation test (CFT), and Enzyme-Linked Immunosorbent Assay (ELISA)—have become the cornerstone of brucellosis surveillance programs due to their relatively low cost, speed, and operational simplicity [8,19,21]. The RBPT is extensively used as a screening test in herds due to its high sensitivity and has been recognized globally [17]. Molecular approaches such as polymerase chain reaction (PCR)-based assays have also demonstrated high sensitivity and species-level differentiation capability, although their routine field application remains limited [18,19]. However, a critical diagnostic challenge persists: despite the wide availability of commercial serological kits, marked variation exists in their performance characteristics, with inconsistent sensitivity, specificity, and reproducibility reported across different manufacturers and settings [16,19,21,22]. Furthermore, cross-reactivity with organisms sharing smooth lipopolysaccharide antigens—most notably Yersinia enterocolitica O:9—and delayed antibody seroconversion in early infection continue to compromise diagnostic accuracy [2,22]. Critically, standardized, head-to-head comparative data evaluating the diagnostic efficacy of different commercial kits under identical field conditions remain scarce, particularly within the context of large-scale ruminant surveillance in regions where brucellosis is endemic. This diagnostic inconsistency represents a fundamental threat to the integrity of epidemiological data: if the measuring instruments themselves are not validated, the disease burden they purport to quantify cannot be trusted.

The objectives of this study were therefore to investigate brucellosis seroprevalence in ruminants (sheep, bovines, and spotted deer) using multiple serological detection approaches—RBPT, STAT, and ELISA—employing three different commercial kits each, and to rigorously compare and evaluate their positive detection rates under standardized conditions.

## 2. Materials and Methods

### 2.1. Samples and Sample Sizes

A total of 5797 serum samples from non-vaccinated ruminants (5260 sheep, 83 bovines, and 454 spotted deer) were used for this study and tested from May 2024 to December 2025. All the serum samples were provided by the State Key Laboratory for Animal Disease Control and Prevention, Lanzhou Veterinary Research Institute.

### 2.2. Serological Tests Used

#### 2.2.1. Rose Bengal Plate Test (RBPT)

All the serum samples were screened using the RBPT three times, with RBPT antigens from three different companies used for the serosurveillance of brucellosis. Owing to a confidentiality agreement with the kit suppliers, the manufacturers’ names are anonymized here as A, B, and C. All the manufacturers have commercially available kits that are widely used in China, and all of them have been granted approval. All three RBPTs utilized in this study employed the *Brucella* S2 strain (CVCC70502) as the antigen. The use of three RBPT antigens in this study aimed to ensure uniformity among the three manufacturers and to compare their ability to detect antibodies for the serosurveillance of brucellosis. All RBPTs were performed according to the standard protocols provided in each manufacturer’s enclosed instructions. RBPT antigens were brought to room temperature and gently shaken to obtain a homogeneous suspension. The test sera were also brought to room temperature 30 min before testing. A total of 25 µL of serum samples to be tested by A and C and 30 µL by B were applied to the RBPT plate. Then, the RBPT antigens to be tested were added in equal volumes to the sera. The sera and RBPT antigens were mixed thoroughly and shaken for four minutes. Results were read carefully for the presence of any agglutinations or clumps under good lighting, by observing with the naked eye, and judged as positive or negative according to the manufacturer’s enclosed instructions. Positive and negative controls were used to validate the reagents utilized and the procedures performed.

#### 2.2.2. Serum Tube Agglutination Test (STAT)

RBPT-positive samples (at least positive for one RBPT) were selected and subjected to the Serum Tube Agglutination Test (STAT) and tested three times using STAT antigens from three different companies used for serosurveillance of brucellosis. STAT company names were also coded as A, B, and C to keep them confidential. All the manufacturers have commercially available kits that are widely used in China; all of them have been granted approval. The STAT antigen used for all three STATs was the *Brucella* S2 strain (CVCC70502). The use of three STAT antigens in this study aimed to ensure uniformity among the three manufacturers, compare their ability to detect antibodies for the serosurveillance of brucellosis, and support the RBPT-positive samples. All STATs were performed according to the standard protocols provided in each manufacturer’s enclosed instructions. STAT antigens were brought to room temperature and gently shaken to obtain a homogeneous suspension. Serum samples were also brought to room temperature 30 min before testing. All the STAT antigens were diluted at a ratio of 1:20 using a normal saline solution before use. All the tested sera were diluted in serial dilutions of 1:12.5, 1:25, 1:50, 1:100, and 1:200, respectively, using the diluted STAT antigens. The negative and positive controls were also set at the same time to validate the reagents used and the procedures performed. Then the test tubes were incubated at 37 °C for 24 h. The results were read carefully for the presence of agglutinates and judged according to the manufacturer’s enclosed instructions. In sheep and spotted deer serum samples, the presence of any agglutinates at the ratio of 1:50 indicated positive outcomes, while the presence of agglutinates at the ratio of 1:25 indicated suspected samples. In bovine serum samples, the presence of any agglutination at the ratio of 1:100 indicated positive results, while the ratio of 1:50 indicated suspected samples.

#### 2.2.3. Enzyme-Linked Immunosorbent Assay (ELISA)

All the samples selected for STATs were also subjected to Enzyme-Linked Immunosorbent Assays (ELISAs) and tested three times using ELISA kits from three different companies. The company names were anonymized as A (indirect ELISA), B (competitive ELISA), and C (competitive ELISA) to maintain confidentiality. All the manufacturers have commercially available kits that are widely used in China; all of them have been granted approval. All three ELISA kits employed *Brucella abortus* lipopolysaccharide (*B. abortus* LPS) as the antigen. The use of three ELISA kits aimed to ensure uniformity among the three manufacturers, compare their ability to detect antibodies for the serosurveillance of brucellosis, and support and increase confidence in the RBPT- and STAT-positive samples. Each ELISA test utilized in this study was performed according to the standard protocols provided in each manufacturer’s enclosed instructions as follows.

**A—indirect ELISA (A i-ELISA):** After bringing all the reagents and samples to be tested to room temperature, 100 μL of sample diluent was added to each well and no diluent was added to the wells for the negative and positive controls. A total of 100 μL of negative and positive controls was added to two wells each, and 10 μL of the samples to be tested was added to the remaining wells. After gentle shaking, the plate was incubated at 37 °C for 30 min. The liquid was emptied and the wells were washed with 300 μL/well working concentration of washing solution five times. The plates were patted on absorbent paper after the last wash. A total of 100 μL of the working concentration of the enzyme-labeled antibody of rabbit anti-sheep IgG was added to each well and incubated at 37 °C for 20 min. The liquid was emptied and the wells were washed with 300 μL/well working concentration of washing solution five times. The plates were patted on absorbent paper after the last wash. A total of 50 μL of the substrate solution A and 50 μL of substrate solution B (substrate solutions A and B can also be mixed in equal proportions to make 100 μL) were added to each well and incubated at 37 °C for 10 min. A total of 50 μL stop solution was added to each well to stop the reaction. Finally, the optical density (OD) value of the samples was read using an ELISA microplate reader with a 450 nm wavelength within 15 min. The cutoff value was calculated by multiplying the average OD value of the negative control by 2.1 (ODNc × 2.1). Test validation was checked by the assessing the values where the OD value of the negative control (ODNc) ≤ 0.4 and the OD value of the positive control (ODPc) ≥ 0.8 (A i-ELISA). Finally, the result was read under the premise that the test was valid. Samples were positive if the OD value of the sample (ODs) ≥ cutoff value, and judged negative if the OD value of the sample (ODs) < cutoff value, based on the manufacturer’s result reading recommendation (A i-ELISA).

**B—competitive ELISA (B c-ELISA):** After bringing all reagents and samples to be tested to room temperature, the samples to be tested, along with the negative and positive controls, were diluted at a ratio of 1:20 using 1% phosphate-buffered saline with tween-20 (PBST) (10 μL of sample + 190 μL of 1% PBST). The ELISA plate was washed once using 300 μL/well of 1% PBST. A total of 50 μL of the diluted serum to be tested, along with the negative and positive controls, was added to the plate (negative and positive controls were set to two wells each). Next, 50 μL of the monoclonal antibody working solution was added to each well. After mixing on a shaker for 5 min, the plate was incubated at 37 °C for 30 min. The liquid was emptied and the wells were washed with 300 μL/well of 1% PBST three times. After drying the plate, 100 μL of sheep anti-mouse enzyme-labeled antibody working solution was added to each well and the plate was incubated at 37 °C for 30 min. The liquid was emptied and the wells were washed with 300 μL/well of 1% PBST three times, and the plate was dried. After mixing substrate solutions A and B in a 1:1 ratio, 100 μL of the mixture was added to each well and incubated at room temperature for 15 min in the dark. Then, 50 μL of stop solution was added to each well to terminate the reaction. Finally, the OD value of the samples was read using an ELISA microplate reader with a 450 nm wavelength within 15 min. The inhibition rate (PI) of the samples was calculated according to the following equation: PI = ((negative control OD450 nm − test sample OD450 nm)/negative control OD450 nm) × 100%. Test validation was checked by the assessing the mean PI of the positive control to be ≥85% (B c-ELISA). Finally, the result was read under the premise that the test was valid. The samples were judged positive for the brucellosis antibody if their PI ≥ 30%, and negative if their PI < 30%, based on the manufacturer’s recommended result reading (B c-ELISA).

**C—competitive ELISA (C c-ELISA):** After bringing all the reagents and the samples to be tested to room temperature, the amount of mixture of the antibody solution and enzyme conjugate was combined in a 1:1 ratio. Bovine serum samples were diluted 5-fold with the sample diluent (20 μL sample + 80 μL sample diluent). Sheep and spotted deer serum samples, along with the negative and positive controls, were not diluted. A total of 50 μL of negative control and 50 μL of the positive control (either bovine or sheep controls, depending on the sample species to be tested) were added to two wells each. Next, 50 μL of the sample to be tested was added to the remaining wells; then, another 50 μL of the enzyme-labeled antibody solution of sheep anti-mouse antibody was added to each well. After mixing well, the plate was incubated at 37 °C for 30 min. After discarding the liquid from each well, the plate was washed with 300 μL/well of wash solution five times. After the last wash, the plate was patted on absorbent paper. A total of 50 μL of the chromogenic solution A and 50 μL of the chromogenic solution B (chromogenic solutions A and B can also be mixed in equal proportions to make 100 μL) were added to each well and incubated at 37 °C for 15 min. Next, 50 μL of stop solution was added to each well to stop the reaction. Finally, the OD value of the samples was read using an ELISA microplate reader with a 450 nm wavelength within 15 min. Test validation was checked by the assessing the positive and negative controls, where the average of the negative control (NCave) − average of the positive control (PCave) ≥ 0.5, and PCave ≤ 0.3 (C c-ELISA). The result determination was calculated by OD450 of sample/OD450 of negative control average (S/Nc). Finally, the result was read under the premise that the test was valid. For bovine samples, if the S/Nc value < 0.5, the sample was judged positive for the bovine *Brucella* antibodies, and if the S/Nc value ≥ 0.5, the sample was judged negative for the bovine *Brucella* antibodies. In sheep and spotted deer, the sample was positive for the *Brucella* antibody if the S/Nc value < 0.3, and negative if the S/Nc value ≥ 0.3, based on the manufacturer’s recommended result reading for each animal species (C c-ELISA).

### 2.3. Statistical Analysis

Positive rates of brucellosis were analyzed by the ratio of the positive results to the total number of animals tested in the Microsoft Excel spreadsheet program. Other descriptive and graphical data were analyzed using IBM SPSS Statistics Version 22 software program. The positivity rates of the three serological tests (RBPT, STAT, and ELISA), along with their kits, was analyzed and compared by assessing the proportion of positive results from the serum samples tested using the Cochran’s Q test in IBM SPSS Statistics.

## 3. Results

### 3.1. Comparative Evaluation of the RBPT Results

A total of 5797 serum samples from ruminants (5260 sheep, 83 bovines, and 454 spotted deer) were screened using RBPT antigens from three companies (A, B, and C). Overall, antigen A yielded the lowest positivity rate, while B and C produced comparable and consistently higher positive rates across all species (Table 1). Notably, antigen A failed to detect any positive cases in bovines or spotted deer, whereas B and C identified positive cases in both species. Full species-level positivity rates are presented in Table 1.

**Figure 1:**
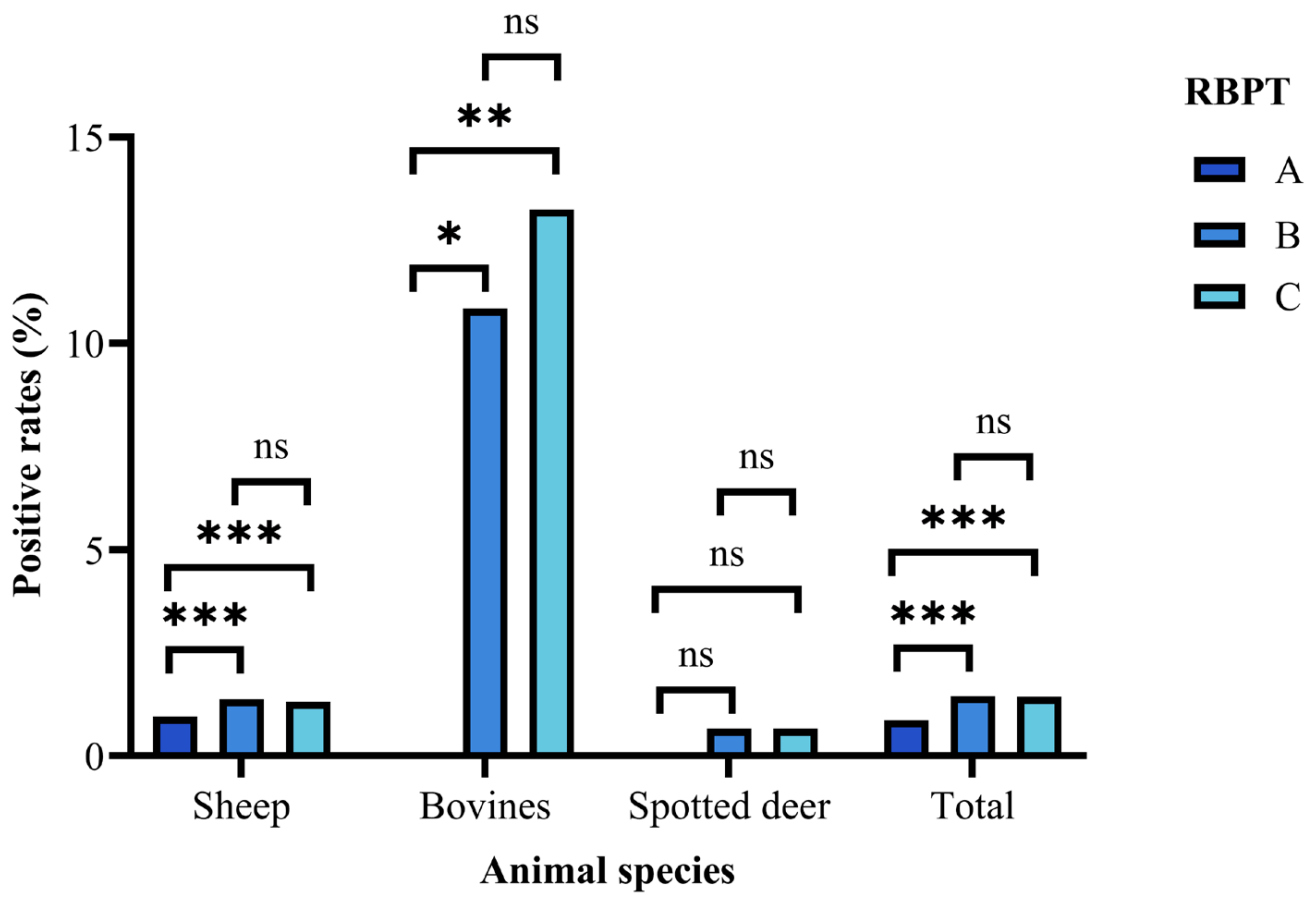
Comparison of the positivity rates of the three RBPT antigens in the three animal species. * *p* < 0.05, ** *p* < 0.01, *** *p* < 0.001, ns: not significant.

Cochran’s Q test revealed a significant difference in positivity rates among the three RBPT antigens overall (Q (2) = 66.06, *p* < 0.001). Follow-up McNemar tests with Bonferroni correction (α = 0.017) showed that antigen A differed significantly from both B and C (both *p* < 0.001), while B and C did not differ from each other (*p* = 1.000) (Figure 1). This pattern was consistent in sheep and bovines (both overall *p* < 0.001), with A detecting significantly fewer positives than B and C, but no significant difference was found between B and C (Figure 1). In spotted deer, no significant difference in detecting positivity rates was observed among the three antigens (Q (2) = 6.00, *p* = 0.05) (Figure 1).

### 3.2. Comparative Evaluation of the STAT Results

Fifty-four RBPT-positive serum samples (40 sheep, 11 bovines, and 3 spotted deer) were further tested using STAT antigens from companies A, B, and C. Overall positivity rates for the RBPT-positive samples were similar across the three antigens, with B yielding the highest positive rate and A the lowest (Table 2). In bovines, all three antigens showed markedly lower positive rates compared to sheep and spotted deer, with A detecting no positives. All spotted deer samples were positive across all three STAT antigens (Table 2).

**Figure 2:**
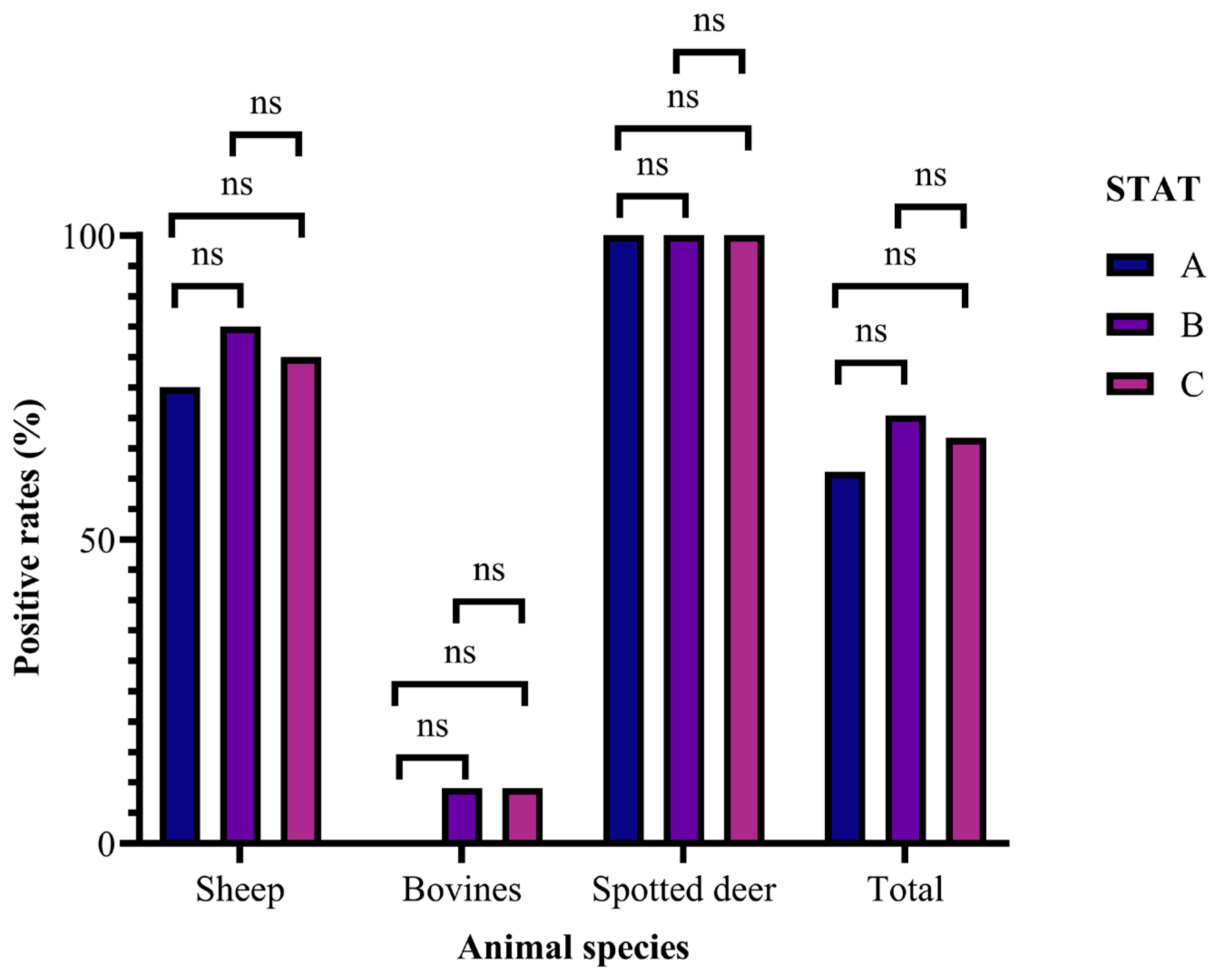
Comparison of the positivity rates of the three STAT antigens in the three selected RBPT-positive animal species. ns: not significant.

Overall, Cochran’s Q test indicated a statistically significant difference in detecting positive rates across the three STAT antigens (Q (2) = 7.60, *p* = 0.022). However, no significant pairwise differences were identified after Bonferroni correction (all *p* > 0.017) (Figure 2). At the species level, statistical testing was limited by small sample sizes, especially for bovines (*n* = 11) and spotted deer (*n* = 3). Sheep (*n* = 40): Cochran’s Q test showed a borderline result (Q (2) = 6.00, *p* = 0.05), suggesting a possible difference in positivity rates among the three STAT antigens, but this did not reach conventional significance (*p* < 0.05). Bovines (*n* = 11): No statistically significant difference was detected (Q (2) = 2.00, *p* = 0.368), but the small sample size limits the reliability of this finding. Descriptively, antigen A detected no positives in bovines, whereas antigens B and C each detected one positive (Table 2). Spotted deer (*n* = 3): All three animals were positive with all three STAT antigens. The identical outcomes preclude formal statistical comparison, and this finding should be interpreted as purely descriptive given the very small sample (Figure 2).

### 3.3. Comparative Evaluation of the ELISA Results

The same 54 selected serum samples were tested using ELISA kits from companies A (i-ELISA), B (c-ELISA), and C (c-ELISA). Overall, kit B achieved the highest positivity rate, while kits A and C were comparable but lower (Table 2). In bovines, kits A and B detected all 11 samples as positive, with kit C detecting slightly fewer (10 samples). Positivity rates in spotted deer were more variable, with kit B reaching 100% and kits A and C each detecting 66.67% (Table 2).

**Figure 3:**
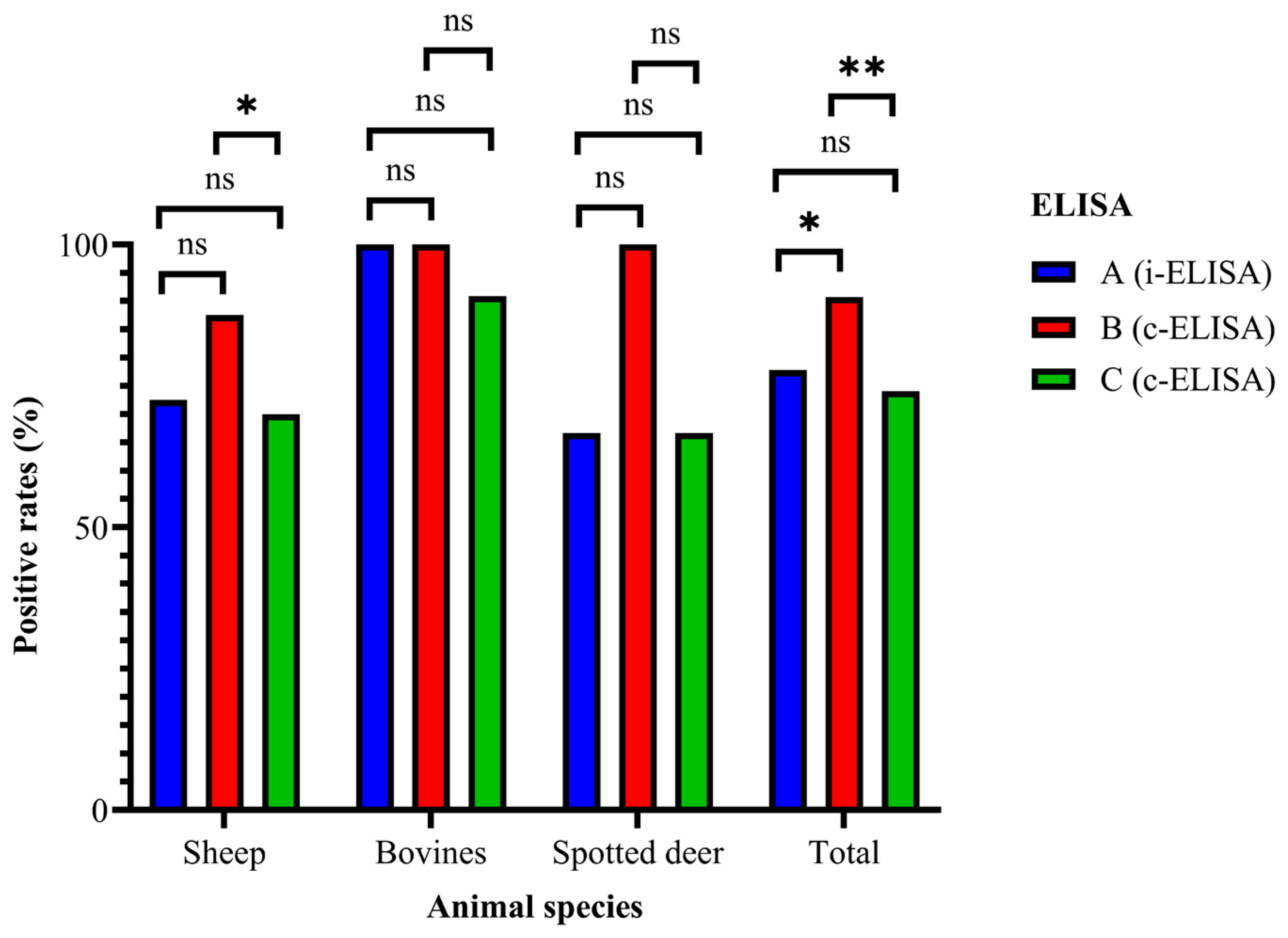
Comparison of the positivity rates of the three ELISA kits in the three selected RBPT-positive animal species. * *p* < 0.05, ** *p* < 0.01, ns: not significant.

Cochran’s Q test indicated a significant difference in positivity rates across the three ELISA kits (Q (2) = 14.89, *p* = 0.001). Pairwise comparisons with Bonferroni correction identified significant differences between A (i-ELISA) and B (c-ELISA) (*p* = 0.016) and between B (c-ELISA) and C (c-ELISA) (*p* = 0.004), but not between A (i-ELISA) and C (c-ELISA) (*p* = 0.500) (Figure 3). In sheep, a significant overall difference was observed (Q (2) = 12.29, *p* = 0.002), driven primarily by the difference between B and C (*p* = 0.016) (Figure 3). In bovines and spotted deer, despite the small sample sizes, no significant differences were found among the three kits (both Q (2) = 2.00, *p* = 0.368) (Figure 3).

### 3.4. Comparison of RBPT, STAT, and ELISA Results

Accurate serological diagnosis of brucellosis depends on tests with high sensitivity and specificity to minimize misclassification of infected and non-infected animals [23]. To assess the relative performance of the three test platforms, Cochran’s Q tests with Bonferroni-corrected McNemar pairwise comparisons were applied to the 54 selected serum samples across all antigen/kit combinations.

#### 3.4.1. Comparative Evaluation of the RBPT and STAT Results

**Figure 4:**
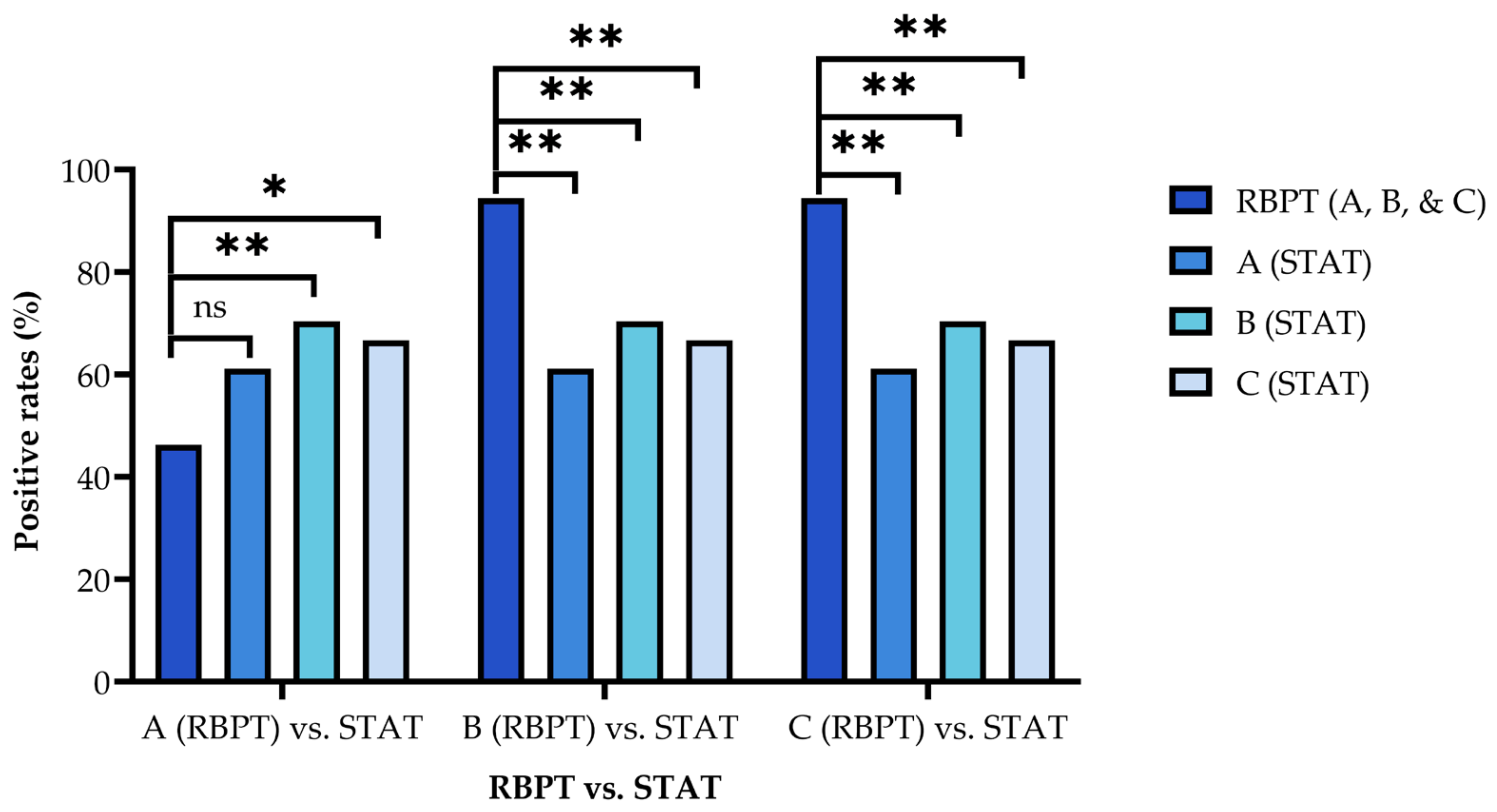
Comparison results of RBPTs and STATs in the total selected RBPT-positive animals. * *p* < 0.05, ** *p* < 0.01, ns: not significant.

For the RBPT-positive samples across all animals, significant overall differences were detected between the RBPT and STAT antigen panels (Q (5) = 85.48, *p* < 0.001). Pairwise comparisons indicated that most RBPT–STAT pairs differed significantly (all *p* < 0.008), with the exception of A (RBPT) vs. A (STAT) (*p* = 0.008) (Figure 4).

At the species level, analyses were limited by small sample sizes for bovines (*n* = 11) and spotted deer (*n* = 3). Statistical results for these subgroups should be interpreted as exploratory rather than definitive. Sheep (*n* = 40): Cochran’s Q test showed a significant overall difference among the six tests (Q (5) = 40.91, *p* < 0.001). Pairwise comparisons with Bonferroni correction identified significant differences between A (RBPT) and B (STAT) and between B (RBPT) and A (STAT) (both *p* = 0.004) (Figure 1 and Figure 2). Bovines (*n* = 11): The small sample size limits reliability of inferential statistics. Descriptively, the pattern of positivity varied across tests, and exploratory Cochran’s Q testing yielded Q (5) = 45.12 (*p* < 0.001). Pairwise comparisons suggested differences involving C (RBPT) versus several STAT antigens (all nominal *p* ≤ 0.004 before correction), but these findings require validation in a larger bovine sample (Figure 1 and Figure 2). Spotted deer (*n* = 3): Given the very small sample (*n* = 3), formal statistical testing is not appropriate. Descriptively, all three animals showed some variation in test positivity, but no reliable inference can be drawn. The exploratory Cochran’s Q result (Q (5) = 15.00, *p* = 0.010) is presented only for completeness and should not be interpreted as evidence of a true difference (Figure 1 and Figure 2).

#### 3.4.2. Comparative Evaluation of the RBPT and ELISA Results

**Figure 5:**
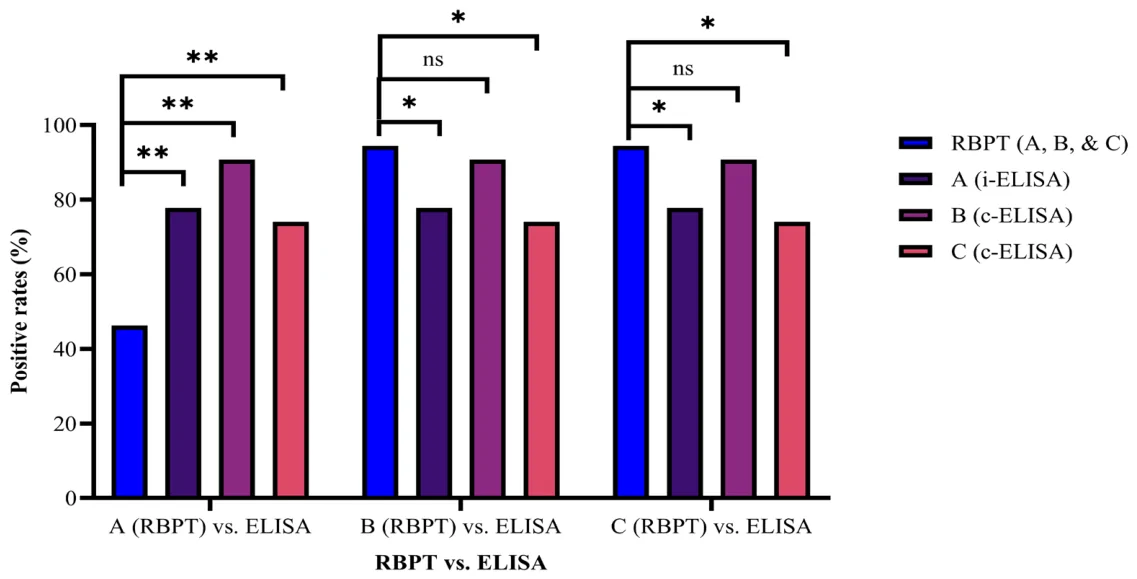
Comparison results of RBPT and ELISA tests in the total selected RBPT-positive animals. * *p* < 0.05, ** *p* < 0.01, ns: not significant.

For overall RBPT-positive samples, significant differences were detected between RBPT and ELISA panels (Q (5) = 87.88, *p* < 0.001). Most pairwise RBPT–ELISA comparisons were significant (all *p* < 0.008), except for B (RBPT) vs. B (c-ELISA) and C (RBPT) vs. B (c-ELISA) (both *p* = 0.500) (Figure 5).

In sheep, the overall difference was significant (Q (5) = 45.68, *p* < 0.001), with significant pairwise differences between A (RBPT) and B (c-ELISA) and B (RBPT) and A (i-ELISA) (both *p* = 0.002), B (RBPT) and C (c-ELISA) (*p* = 0.001), and C (RBPT) and C (c-ELISA) (*p* = 0.004). In bovines, descriptively, overall differences were also observed (Q (5) = 45.16, *p* < 0.001), primarily driven by A (RBPT) differing from all three ELISA kits (all *p* ≤ 0.002). In spotted deer, descriptively, no difference was observed (Q (5) = 10.79, *p* = 0.056) (Figure 1 and Figure 3).

#### 3.4.3. Comparative Evaluation of the STAT and ELISA Results

**Figure 6:**
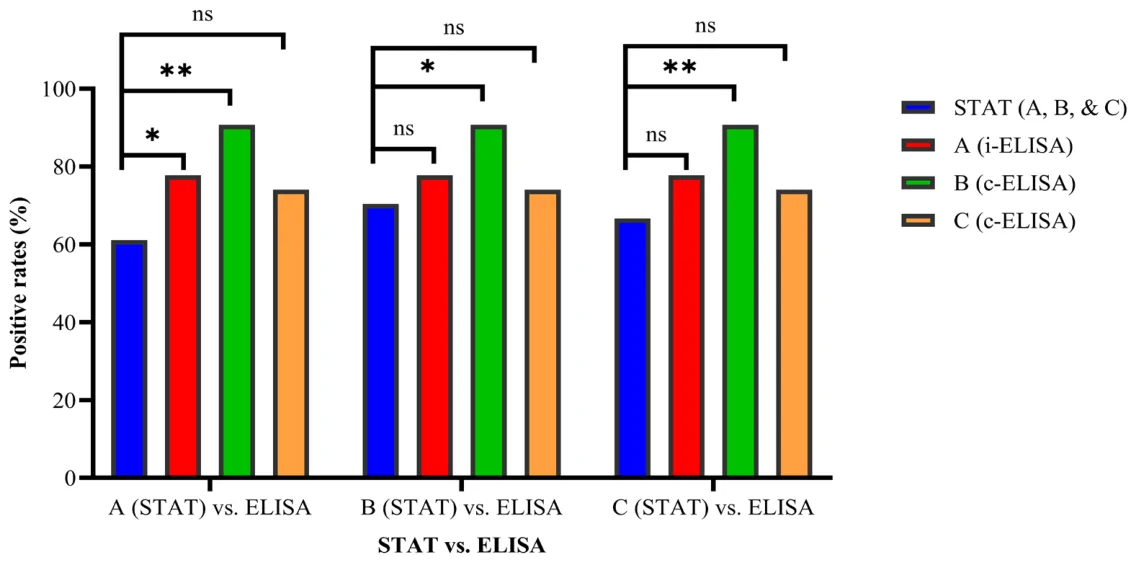
Comparison results of STATs and ELISAs in the total selected RBPT-positive animals. * *p* < 0.05, ** *p* < 0.01, ns: not significant.

For overall RBPT-positive samples, significant differences were detected between STAT and ELISA test panels (Q (5) = 46.00, *p* < 0.001). Significant pairwise differences were identified between A (STAT) and A (i-ELISA) (*p* = 0.004), A (STAT) and B (c-ELISA) and C (STAT) and B (c-ELISA) (both *p* < 0.001), and B (STAT) and B (c-ELISA) (*p* = 0.001), while all other pairs did not differ significantly (all *p* > 0.008) (Figure 6).

At the species level, a significant overall difference was observed in sheep (Q (5) = 22.69, *p* < 0.001), though no individual pairwise comparison reached significance after Bonferroni correction (all *p* > 0.008). In bovines, descriptively, overall differences were suggested (Q (5) = 48.30, *p* < 0.001), for all pairwise STAT–ELISA comparisons (all nominal *p* < 0.008). In spotted deer, descriptively, no differences were detected (Q (5) = 5.00, *p* = 0.416) (Figure 2 and Figure 3).

## 4. Discussion

Serological tests have been used singly or in combination to detect the prevalence of *Brucella* infection [24,25]; however, their positivity rates vary considerably across manufacturers, animal species, and epidemiological contexts, underscoring the need for continuous and rigorous comparative evaluation.

In this study, RBPT antigens from companies B and C demonstrated comparable positivity rates, with no statistically significant difference in seroprevalence estimates (1.45% vs. 1.43%, *p* > 0.05) across overall samples, sheep, and bovines. In contrast, company A’s RBPT antigen consistently detected lower positives, yielding a seroprevalence of only 0.86%—approximately 40% lower than that of companies B and C—with statistically significant differences confirmed in overall samples, sheep, and bovines (*p* < 0.05). Notably, no significant difference was detected among the three RBPT antigens in spotted deer (*p* = 0.05), possibly reflecting the small sample size of this species, which limits statistical power.

Although all three RBPT antigens are derived from the S2 strain, the observed performance gap attributable to company A warrants mechanistic scrutiny. Potential explanations include insufficient antigen concentration, suboptimal buffer pH formulation, or inadequate quality control (QC) during manufacturing. Since the RBPT relies on agglutination reactions driven by antigen–antibody binding avidity, even subtle differences in antigen density or suspension stability can substantially alter test sensitivity. These technical discrepancies among commercially available kits have been insufficiently explored in the literature and deserve dedicated investigation.

From a disease control perspective, the practical implications of using a lower-performing RBPT kit are severe. An estimated 40% of truly seropositive animals could be missed when company A’s kit is used relative to companies B or C (if companies B and C are correct). In field surveillance settings, such false negatives would allow infected animals to remain undetected within herds, facilitating continued transmission of brucellosis—an outcome with potentially catastrophic consequences for both animal health and human public health. There could also be a result of culling an estimated 40% of false seropositive animals if kit A is correct. These findings strongly argue against the interchangeable use of RBPT kits from different manufacturers without prior independent validation.

The RBPT positivity rates in sheep (0.95%, 1.37%, and 1.31% for companies A, B, and C, respectively) were broadly consistent with the pooled national seroprevalence of 1.00% (95% CI: 0.70–1.30) reported between 2000 and 2009 in China, though lower than the more recent estimates of 2.30–3.20% [26]. This temporal discrepancy likely reflects improved control measures over time, although regional variation cannot be excluded. In bovines, company A again yielded anomalously low estimates (0%), while companies B and C showed seroprevalences of 10.84% and 13.25%, respectively—both substantially exceeding the national pooled estimate of 1.5% (95% CI: 0.6–2.6%) [27] and the dairy cattle estimate of 1.9% [28]. The marked elevation in bovine seroprevalence detected by companies B and C may reflect genuine regional endemicity, or may partly result from false positives; this ambiguity further supports the need for confirmatory testing. In spotted deer, seroprevalence was 0% (company A) and 0.66% (companies B and C), lower than the 3.6% [29] for red deer, though direct comparisons across cervid species must be made cautiously.

Unlike the RBPT, the three STAT antigens from companies A, B, and C demonstrated no statistically significant differences in positivity rates across all animal species of RBPT-positive samples, with positivity rates of 61.11%, 70.37%, and 66.67%, respectively, among RBPT-positive samples (all *p* > 0.017 for pairwise comparisons). This inter-manufacturer consistency is reassuring from a standardization standpoint; however, STATs consistently yielded lower positive rates than ELISAs across all three manufacturers. This observation aligns with the well-established understanding that the STAT, while historically important, is technically demanding, subjective in endpoint reading, and susceptible to false negatives in chronic or low-titer infections. These characteristics limit its utility as a standalone detection test in modern surveillance programs.

Though not supported by confirmatory tests, all three ELISA kits demonstrated higher positive rates (77.78%, 90.74%, and 74.07% for companies A, B, and C, respectively) than the corresponding STATs among RBPT-positive samples, confirming the superior sensitivity of ELISA for detecting *Brucella* antibodies. Despite the sample differences (whole samples and subset of RBPT-positive samples), those results are consistent with the overall brucellosis seroprevalences of 33.85%, 32.60% and 30.90%, respectively, by the ELISA, RBPT and STAT [24], and bovine prevalence rates of 74.3%, 83.7%, and 81.3% by the CFT, i-ELISA, and RBPT [30], respectively. However, significant inter-kit variability was observed: company B’s c-ELISA produced substantially higher positive rates than company C’s c-ELISA and company A’s i-ELISA, with statistically significant pairwise differences (B vs. C: *p* = 0.004; A vs. B: *p* = 0.016). This is particularly noteworthy given that companies B and C both employ competitive-ELISA formats; the divergence between them likely reflects differences in conjugate quality, blocking efficiency, or antigen coating density rather than fundamental methodological differences.

The consistently higher sensitivity of ELISA relative to the STAT supports a detection rate algorithm in which ELISA replaces the STAT as the preferred serosurveillance test following RBPT initial screening. The data from this study indicate that relying on the STAT as a second-tier test risks missing a clinically and epidemiologically meaningful proportion of true positives that would be captured by ELISA. The strong agreement between the RBPT and ELISA, corroborated by multiple prior studies reporting Kappa values ranging from 0.868 (cattle) to 0.998 (goats) [23], excellent agreement in cattle (0.8597) [24], and excellent agreement (Kappa = 0.8847) [25], further supports the adoption of RBPT-plus-ELISA as the recommended two-step serosurveillance framework.

There are several limitations recognized in this study. First, only RBPT-positive samples were subsequently tested by the STAT and ELISA. This design, while pragmatically efficient, means that the sensitivity of the STAT and ELISA cannot be fully evaluated, as RBPT-negative but ELISA-positive animals (potential false negatives of the RBPT) were not identified. Therefore, the inter-serological test comparison is not fully representative. Future studies incorporating ELISA screening of the full sample cohort would provide a more complete picture of true seroprevalence and enable a more rigorous assessment of each test’s sensitivity and specificity. Second, the absence of gold-standard bacteriological confirmation limits the interpretation of discordant serological results. Third, due to the small number of bovine (*n* = 11) and spotted deer (*n* = 3) samples, species-level statistical comparisons are underpowered and prone to instability. Accordingly, we emphasize descriptive patterns over *p*-values for these subgroups, and our conclusions are framed cautiously. Our study reveals significant variability in the detection performance of commercial RBPT and ELISA kits. The choice of manufacturer can significantly alter seroprevalence estimates. Rigorous validation of kits is essential before national surveillance. Furthermore, ELISA showed higher positive rates than the STAT for the RBPT-positive cases. Fourth, all the diagnostic kits were labeled for bovines and small ruminants (sheep and goats). Because no diagnostic kits are specifically designed for spotted deer, the study used kits intended for small ruminants (sheep and goats) instead. This approach may introduce diagnostic limitations when testing spotted deer.

## 5. Conclusions

Although several methods, including the RBPT, STAT, and ELISA, exist, the positivity rates among the kits vary significantly across manufacturers. Due to this inconsistency and the general lack of standardized comparison data for veterinary diagnostic reagents worldwide—including in China—the study’s findings highlight the need to evaluate different kits, as the current situation risks errors in diagnosing and interpreting epidemiological data.

## Figures and Tables

**Table 1:** Comparison of the three RBPT results for all animals tested.

RBPT Antigens	Animal Species	Total Samples Tested	Positives	Positive %
A	Sheep	5260	50	0.95
	Bovine	83	0	0.00
	Spotted deer	454	0	0.00
	Total	5797	50	0.86
B	Sheep	5260	72	1.37
	Bovine	83	9	10.84
	Spotted deer	454	3	0.66
	Total	5797	84	1.45
C	Sheep	5260	69	1.31
	Bovine	83	11	13.25
	Spotted deer	454	3	0.66
	Total	5797	83	1.43

**Table 2:** Comparison of RBPT, STAT, and ELISA results for the selected serum samples.

Animal Species	Total No. of Selected Samples	RBPT Results			STAT Results			ELISA Results		
		A Positive No. (%)	B Positive No. (%)	C Positive No. (%)	A Positive No. (%)	B Positive No. (%)	C Positive No. (%)	A (i-ELISA) Positive No. (%)	B (c-ELISA) Positive No. (%)	C (c-ELISA Positive No. (%)
Sheep	40	25 (62.5)	39 (97.5)	37 (92.5)	30 (75)	34 (85)	32 (80)	29 (72.50)	35 (87.50)	28 (70)
Bovine	11	0 (0)	9 (81.82)	11 (100)	0 (0)	1 (9.09)	1 (9.09)	11 (100)	11 (100)	10 (90.91)
Spotted deer	3	0 (0)	3 (100)	3 (100)	3 (100)	3 (100)	3 (100)	2 (66.67)	3 (100)	2 (66.67)
Total	54	25 (46.27)	51 (94.44)	51 (94.44)	33 (61.11)	38 (70.37)	36 (66.67)	42 (77.78)	49 (90.74)	40 (74.07)

## Data Availability

The original contributions presented in this study are included in the article. Further inquiries can be directed to the corresponding authors.

## Abbreviations

The following abbreviations are used in this manuscript:

RBPT	Rose Bengal Plate Test
STAT	Serum Tube Agglutination Test
ELISA	Enzyme-Linked Immunosorbent Assay
WHO	World Health Organization
FAO	Food and Agriculture Organization
WOAH	World Organization for Animal Health
CFT	Complement Fixation Test
PCR	Polymerase Chain Reaction
i-ELISA	Indirect ELISA
c-ELISA	Competitive ELISA
OD	Optical Density
ODNc	OD Value of the Negative Control
ODPc	OD Value of the Positive Control
ODs	OD Value of the Sample
PBST	Phosphate-Buffered Saline with Tween-20
PI	Inhibition Rate
NCave	Average of the Negative Control
PCave	Average of the Positive Control
S/Nc	Sample over Negative Control Average
QC	Quality Control
